# Rates of Adverse Events in Patients With Ulcerative Colitis Undergoing Colectomy During Treatment With Tofacitinib vs Biologics: A Multicenter Observational Study

**DOI:** 10.14309/ajg.0000000000002676

**Published:** 2024-02-02

**Authors:** Gabriele Dragoni, Tommaso Innocenti, Aurelién Amiot, Fabiana Castiglione, Laura Melotti, Stefano Festa, Edoardo Vincenzo Savarino, Marie Truyens, Konstantinos Argyriou, Daniele Noviello, Tamas Molnar, Vincent Bouillon, Cristina Bezzio, Piotr Eder, Samuel Fernandes, Anna Kagramanova, Alessandro Armuzzi, Raquel Oliveira, Anna Viola, Davide Giuseppe Ribaldone, Ioannis Drygiannakis, Chiara Viganò, Francesca Calella, Antonietta Gerarda Gravina, Daniela Pugliese, María Chaparro, Pierre Ellul, Sophie Vieujean, Monica Milla, the “TOFA-poSTOP” Study Group, Flavio Caprioli

**Affiliations:** 1 IBD Referral Centre, Careggi University Hospital, Florence, Italy;; 2 Department of Experimental and Clinical Biomedical Sciences “Mario Serio,” University of Florence, Florence, Italy;; 3 Department of Gastroenterology, Henri Mondor University Hospital, Paris Est-Creteil University, Creteil, France;; 4 Department of Clinical Medicine and Surgery, “Federico II” University of Naples, Naples, Italy;; 5 Department of Medical and Surgical Sciences, Alma Mater Studiorum University of Bologna, Bologna, Italy;; 6 IBD Unit, “San Filippo Neri' Hospital, Rome, Italy;; 7 Department of Surgery, Oncology and Gastroenterology, University of Padova, Padova, Italy;; 8 Department of Gastroenterology, Ghent University Hospital, Ghent, Belgium;; 9 Department of Gastroenterology, University Hospital of Larisa, Larissa, Greece;; 10 Department of Pathophysiology and Transplantation, Università degli Studi di Milano, Milan, Italy;; 11 Department of Gastroenterology, Albert Szent-Györgyi Medical School, University of Szeged, Szeged, Hungary;; 12 Department of Gastroenterology, Erasme University Hospital, Brussels, Belgium;; 13 IBD Center, IRCCS Humanitas Research Hospital, Rozzano, Milan, Italy;; 14 Department of Gastroenterology, Dietetics and Internal Medicine–Poznań University of Medical Sciences, Heliodor Święcicki University Hospital, Poznań, Poland;; 15 Department of Gastroenterology and Hepatology, Hospital de Santa Maria, Centro Hospitalar Universitário de Lisboa Norte, Lisboa, Portugal;; 16 Clínica Universitária da Faculdade de Medicina de Lisboa, Lisboa, Portugal;; 17 Moscow Clinical Scientific Center named after A.S. Loginov, Moscow, Russian Federation;; 18 Gastroenterology Department, Algarve University Hospital Centre–Portimão Unit, Algarve, Portugal;; 19 IBD-Unit, Department of Clinical and Experimental Medicine, University of Messina, Messina, Italy;; 20 Division of Gastroenterology, Department of Medical Sciences, University of Turin, Turin, Italy;; 21 Department of Gastroenterology, University Hospital of Heraklion, Heraklion, Greece;; 22 Division of Gastroenterology, Center for Autoimmune Liver Diseases, Department of Medicine and Surgery, University of Milano-Bicocca, Monza, Italy;; 23 European Reference Network on Hepatological Diseases (ERN RARE-LIVER), Fondazione IRCCS San Gerardo dei Tintori, Monza, Italia;; 24 SOC Gastroenterologia ed endoscopia digestiva, Azienda USL Toscana Centro, Ospedale “San Giuseppe,” Empoli, Italy;; 25 Hepatogastroenterology Unit, Department of Precision Medicine, University of Campania “Luigi Vanvitelli,” Naples, Italy;; 26 CEMAD, IBD Unit, Dipartimento di Scienze Mediche e Chirurgiche, Fondazione Policlinico Universitario “A. Gemelli” IRCCS, Rome, Italy;; 27 Gastroenterology Unit, Hospital Universitario de La Princesa, Instituto de Investigación Sanitaria Princesa (IIS-Princesa), Universidad Autónoma de Madrid (UAM), Centro de Investigación Biomédica en Red de Enfermedades Hepáticas y Digestivas, Madrid, Spain;; 28 Department of Medicine, Division of Gastroenterology, Mater Dei Hospital, Msida, Malta;; 29 Hepato-Gastroenterology and Digestive Oncology, University Hospital CHU of Liège, Liège, Belgium;; 30 Gastroenterology and Endoscopy Unit, Fondazione IRCCS Cà Granda, Ospedale Maggiore Policlinico di Milano, Milan, Italy.

**Keywords:** colectomy, postoperative safety, tofacitinib, ulcerative colitis

## Abstract

**INTRODUCTION:**

Patients with ulcerative colitis (UC) receiving immunosuppressive drugs are at substantial risk of colectomy. We aimed to assess the risk of postoperative complications of tofacitinib exposure before colectomy in comparison with biologics.

**METHODS:**

A multicenter, retrospective, observational study was conducted in patients with UC who underwent total colectomy for medically refractory disease, exposed to tofacitinib or a biologic before surgery. Primary outcome was the occurrence of any complication within 30 (early) and 90 (late) days after surgery. Secondary outcomes were the occurrence of infections, sepsis, surgical site complications, venous thromboembolic events (VTE), hospital readmissions, and redo surgery within the same timepoints.

**RESULTS:**

Three hundred one patients (64 tofacitinib, 162 anti-tumor necrosis factor-α agents, 54 vedolizumab, and 21 ustekinumab) were included. No significant differences were reported in any outcome, except for a higher rate of early VTE with anti-tumor necrosis factor-α agents (*P* = 0.047) and of late VTE with vedolizumab (*P* = 0.03). In the multivariate analysis, drug class was not associated with a higher risk of any early and late complications. Urgent colectomy increased the risk of any early (odds ratio [OR] 1.92, 95% confidence interval [CI] 1.06–3.48) complications, early hospital readmission (OR 4.79, 95% CI 1.12–20.58), and early redo surgery (OR 7.49, 95% CI 1.17–47.85). A high steroid dose increased the risk of any early complications (OR 1.96, 95% CI 1.08–3.57), early surgical site complications (OR 2.03, 95% CI 1.01–4.09), and early redo surgery (OR 7.52, 95% CI 1.42–39.82). Laparoscopic surgery decreased the risk of any early complications (OR 0.54, 95% CI 0.29–1.00), early infections (OR 0.39, 95% CI 0.18–0.85), and late hospital readmissions (OR 0.34, 95% CI 0.12–1.00).

**DISCUSSION:**

Preoperative tofacitinib treatment demonstrated a postoperative safety profile comparable with biologics in patients with UC undergoing colectomy.

## Graphical abstract

**Figure:**
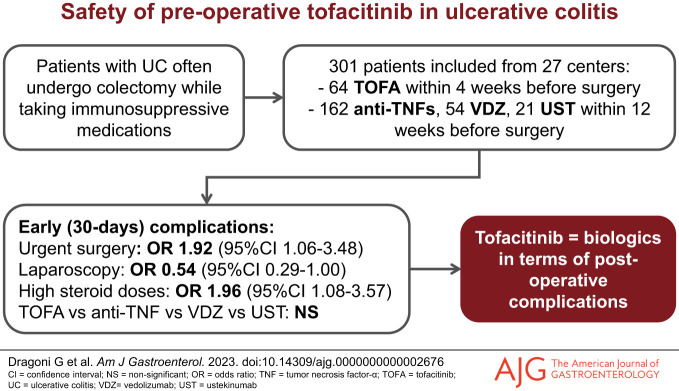

## BACKGROUND

Ulcerative colitis (UC) is a chronic inflammatory bowel disease (IBD), characterized by relapsing and remitting mucosal inflammation, usually starting in the rectum and extending to the proximal segments of the colon (1). Despite the increasing number of available medical options for the treatment of UC (2), the risk of undergoing proctocolectomy has only slightly decreased over the years (3) with up to 15% of patients with UC still requiring surgery during their lifetime (1).

Tofacitinib is the first oral Janus kinase (JAK) inhibitor small molecule approved for the treatment of moderate-to-severe UC (4). Tofacitinib blocks several cytokine cascades by inhibiting the tyrosine kinases of the JAK/signal transducer and activator of transcription pathway (5). Previous studies on rheumatoid arthritis raised safety concerns regarding increased episodes of venous thromboembolic events (VTE) and major cardiovascular events (6), leading the American and European medical agencies to suggest caution in the use of JAK inhibitors in chronic inflammatory disorders (7,8). However, recent meta-analyses in patients with IBD have not confirmed this additional risk, particularly when tofacitinib was compared with other biologics and small molecules (9,10). In the surgical setting, tofacitinib has been marginally investigated regarding the safety of preoperative administration and the potential need for a washout period before surgery. Total colectomy is often required while patients with UC are still receiving immunosuppressive drugs, and it might be controversial to define the safe timing of surgery based on the last administration of these medications.

To date, only one small retrospective study on 53 patients with UC undergoing colectomy has assessed the postoperative outcomes of patients treated with tofacitinib in the preoperative phase. This study reported an overall surgical infective complication rate comparable with biologics (11%), with a possible increased risk of postoperative VTE (13%) (11). Because robust evidence is lacking, we developed a multicenter study aiming to assess the risk of complications of tofacitinib exposure before colectomy in comparison with anti–tumor necrosis factor-α (TNF) agents, vedolizumab, and ustekinumab, in patients with medically refractory UC.

## METHODS

### Study design

A multicenter, retrospective, observational study was conducted in 27 centers across Europe. The study was approved by the Ethical Committee of the coordinating center (Careggi University Hospital, Florence, Italy) on November 8, 2022 (Ref CEAVC-22824). The study protocol conforms to the ethical guidelines of the 1975 Declaration of Helsinki. Every participating center independently uploaded patient data in specific electronic case report forms using Research Electronic Data Capture platform (12) hosted at the University of Florence (Florence, Italy).

Patients with active UC and ongoing medication referred to a total colectomy because of medically refractory disease between September 2005 and February 2023 were included in the study. The following groups of immunosuppressive drugs were considered as active treatment: tofacitinib, anti-TNFs (infliximab, adalimumab, and golimumab), vedolizumab, and ustekinumab. Inclusion criteria were the following: established diagnosis of UC at the time of inclusion; medically refractory UC as reason for surgery; administration of the last dose of tofacitinib within 4 weeks of colectomy; or administration of the last dose of biologics within 12 weeks before colectomy. The intervals between the last administration of drugs and surgery have been derived from the existent literature in the field (11,13) to allow for comparisons and future data pooling. Exclusion criteria included age <18 years; inability to provide written informed consent; diagnosis of indeterminate colitis; colectomy performed for intestinal cancer; concomitant use of more than one biologic or combination with immunosuppressants; use of more than 1 immunomodulator within 12 weeks before colectomy; and inability to document the first 90 days of postsurgical follow-up. Patients were then divided in 4 groups based on the medication administered in the preoperative phase, with all anti-TNF treatment considered in the same group.

Demographic and clinical covariates that were considered in the analyses were the following: sex; age at diagnosis; age at surgery; disease duration at surgery; drug exposure; history of immunosuppressive drugs (experienced vs naive to biologics); history of acute severe ulcerative colitis; disease extension at surgery (Montreal classification (14)); active smoking; Charlson Comorbidity Index (15); diabetes mellitus; peripheral vascular disease; body mass index at surgery (kg/m^2^); concomitant treatment with corticosteroids (yes/no, and if yes < or > 20pmg/d oral prednisone equivalent for the 4 weeks before surgery); use of prophylactic antibiotics in the week after surgery (yes/no); use of prophylactic low-molecular-weight heparin (LMWH) in the week after surgery (yes/no); type of surgery (elective vs urgent; laparotomy vs laparoscopy; colectomy + ileostomy vs proctocolectomy + ileal pouch-anal anastomosis [IPAA] + ileostomy vs one-stage proctocolectomy + IPAA). Data regarding clinical activity (with partial Mayo score (16)), endoscopic activity (with Mayo endoscopic subscore (17)), and laboratory results (hemoglobin [g/dL], leukocytes [×10^9^/L], platelets [×10^9^/L], albumin [g/dL], and C-reactive protein [mg/dL] within 2 weeks before surgery) were also collected. A shorter time interval between the last administration of drug and surgery (7 days for tofacitinib and 8 weeks for biologics) was also considered as a covariate.

### Outcomes

The primary outcome of the study was the occurrence of any postoperative complications within 30 days (defined as “early”) and 90 days (defined as “late”) after surgery between patients with exposure to tofacitinib and patients with exposure to one of the 3 groups of biologics, i.e., anti-TNFs, vedolizumab, or ustekinumab. The secondary outcomes of the study were the occurrence of infections (urinary-tract infections, pneumonia, bacteremia, or other general infections), sepsis, surgical site complications (SSC), VTE, unplanned hospital readmissions, redo surgery, and death within the same predefined timepoints.

Sepsis was defined following the criteria of the Sepsis-3 consensus (18). SSC included superficial surgical site infection (SSI), suture line leak, delayed wound healing, abdominal or pelvic abscess, anastomotic (or rectal stump) leak, and obstruction at the site of anastomosis.

### Statistical analysis

Continuous variables were expressed as medians and interquartile ranges (IQR) or mean values and SDs when appropriate. Categorical variables were presented as percentages. After normality was assessed, homogeneity between the study groups and univariate analysis for 30- and 90-day outcomes was tested with the χ^2^ test for categorical variables, whereas independent-samples *t* test or Kruskal–Wallis test and Mann–Whitney *U* test were used for normally or non-normally distributed continuous variables, respectively. A 2-sided *P* value < 0.05 was considered significant. Relevant variables (*P* < 0.1 at univariate analysis) were analyzed with a multivariable model by using binary logistic regression to minimize the effect of potential confounding factors, wherever feasible considering the number of events per outcome. The reference drug in the multivariable model was tofacitinib. Odds ratios (OR) were expressed with a 95% confidence interval (CI). Because most variables presented less than 10% of missing data, multiple imputation approach was considered unnecessary. Variables with more than 10% of missing data (i.e., clinical and endoscopic disease activity) were not included in the multivariable model. All statistical analyses were performed with SPSS Statistics version 25.0 (IBM, Armonk, NY).

## RESULTS

Medical records of 327 patients were reviewed, of which 301 included in the final analysis after excluding 26 patients who stopped the study drug before colectomy earlier than allowed. Among the total population, 64 (21.3%) patients were in the tofacitinib group, whereas 237 were on biologics: 162 (53.8%), 54 (17.9%), and 21 (7.0%) in the anti-TNF, vedolizumab, and ustekinumab groups, respectively. Administration of the last dose of tofacitinib occurred at a median time of 13 days (IQR 4–20) before surgery, whereas last dose of anti-TNF, vedolizumab, and ustekinumab was administered at a median of 21 (IQR 14–38), 34 (IQR 18–50), and 27 (IQR 16–31) days before surgery, respectively. In total, 236 patients (78.4%) had their last drug dose within a shorter time before surgery: 22 (34.4%) tofacitinib within 7 days, with 148 (91.4%) anti-TNF, 46 (85.2%) vedolizumab, and 20 (95.2%) ustekinumab within 8 weeks. Baseline and demographic characteristics of the cohort are reported in Table 1. A higher percentage of patients in the anti-TNF group underwent urgent surgery compared with the other 3 groups. Median age at surgery was higher in the vedolizumab and ustekinumab groups than in the anti-TNF and tofacitinib groups, with a higher median Charlson Comorbidity Index in vedolizumab and ustekinumab groups because of an increased prevalence of comorbidities. Clinical and endoscopic activity was available for 276 (91.7%) and 256 (85.0%) patients, respectively, with homogeneous characteristics across groups (Table 1). No patients died during the 90-day observation period after surgery.

**Table 1.**
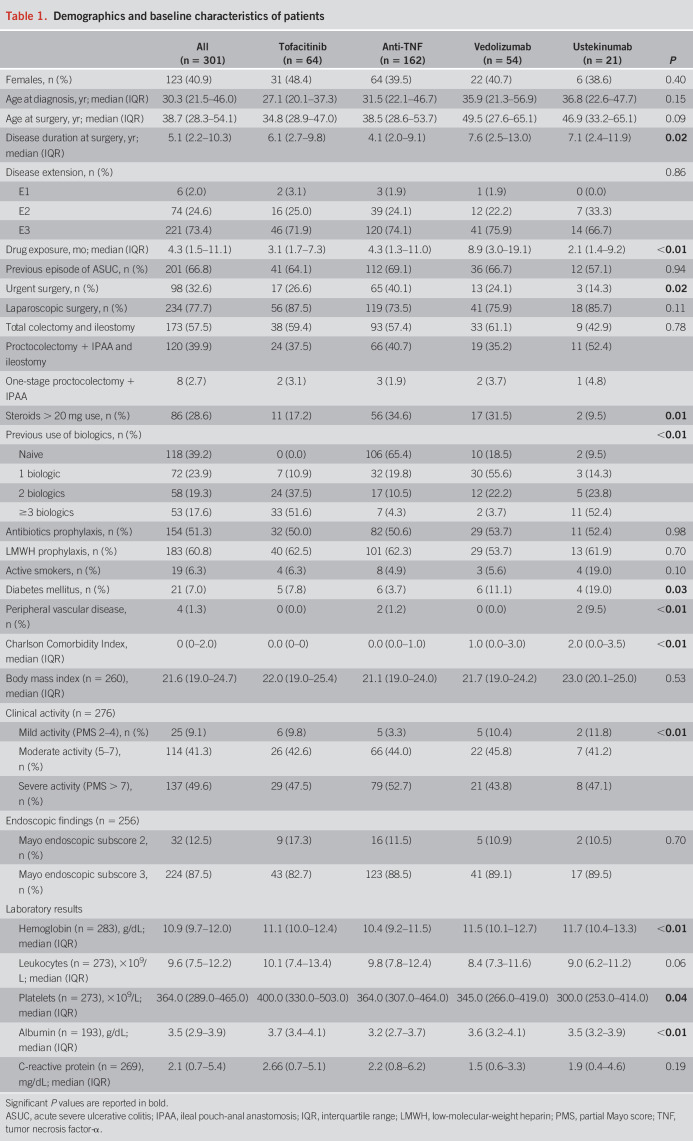
Demographics and baseline characteristics of patients

In total, 90 (29.9%) patients had at least one early complication, whereas 26 (8.5%) had at least one late complication between 30 and 90 days after surgery. Early complications were reported as follow: 40 (13.3%) infections, 17 (5.6%) sepsis, 53 (17.6%) SSC, 9 (3.0%) VTE, 26 (8.6%) hospital readmission, and 20 (6.6%) redo surgery. Among the 40 early infections, 16 were urinary tract infections, 7 pneumonia, 6 bacteremia, and 11 other general infections, including one case of documented SARS-CoV-2 infection without pneumonia. Among the early SSC, 12 patients had a superficial SSI, 13 a suture line leak, 4 a delayed wound healing, 11 an abdominal or pelvic abscess, 9 an anastomotic leak, and 4 an obstruction at the site of anastomosis. As late complications, 5 (1.7%) infections, 1 (0.3%) sepsis, 11 (3.6%) SSC, 2 (0.7%) VTE, 17 (5.6%) hospital readmissions, and 4 (1.3%) redo surgeries were reported. Among late infections, 1 patient had a urinary tract infection, 1 had a pneumonia, and 3 had other unspecified infections. Regarding late SSC, 2 patients had a superficial SSI, 2 had a suture line leak, 4 had a delayed wound healing, and one event each occurred for abdominal abscess, anastomotic leak, and obstruction at the site of anastomosis.

Detailed information regarding the outcomes is reported in Tables 2–4 and Table 5. There were no statistically significant differences in the occurrence of any early (*P* = 0.07) and late (*P* = 0.67) complications between the 4 groups of drugs. Among the secondary outcomes, there were no differences between the 4 groups in terms of early (*P* = 0.31) and late (*P* = 0.50) infections, early (*P* = 0.10) and late (*P* = 0.29) sepsis, early (*P* = 0.40) and late (*P* = 0.19) SSC, early (*P* = 0.82) and late (*P* = 0.70) hospital readmissions, and early (*P* = 0.43) and late (*P* = 0.46) redo surgeries. All 9 early VTE occurred in the anti-TNF group (*P* = 0.047), whereas the 2 late VTE occurred in the vedolizumab group (*P* = 0.03). Notably, 5 of 9 early VTE occurred in patients on prophylactic LMWH, whereas none of the 2 patients with late VTE was administered LMWH. However, VTE were generally rare, and no events were recorded in the tofacitinib group. Consistent results were found after restricting for both shorter exposure windows and year of surgery (see Supplementary Tables S1–S3, http://links.lww.com/AJG/D177).

**Table 2.**
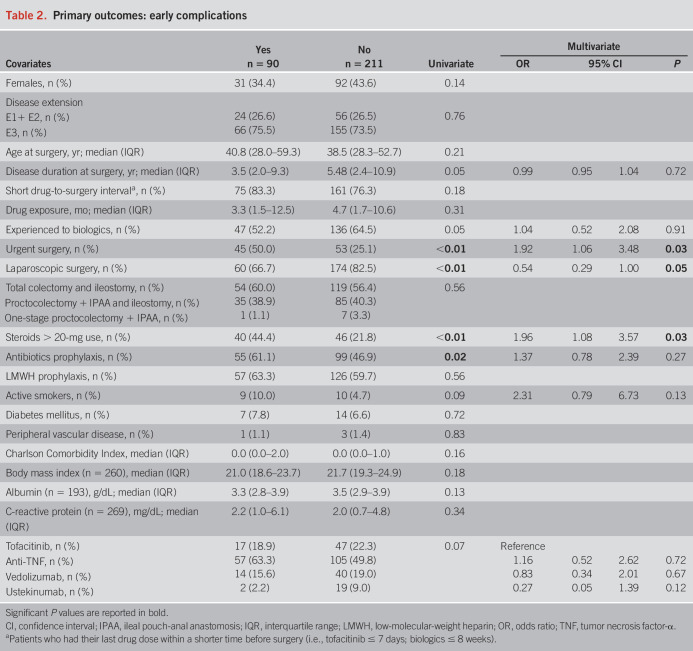
Primary outcomes: early complications

**Table 3.**
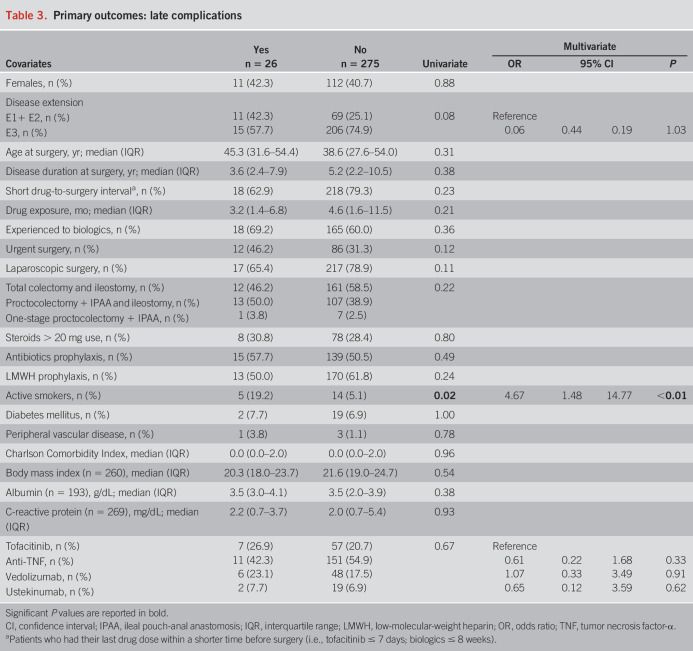
Primary outcomes: late complications

**Table 4.**
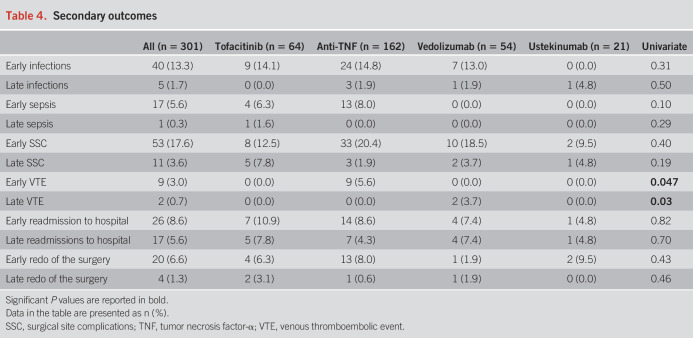
Secondary outcomes

**Table 5.**
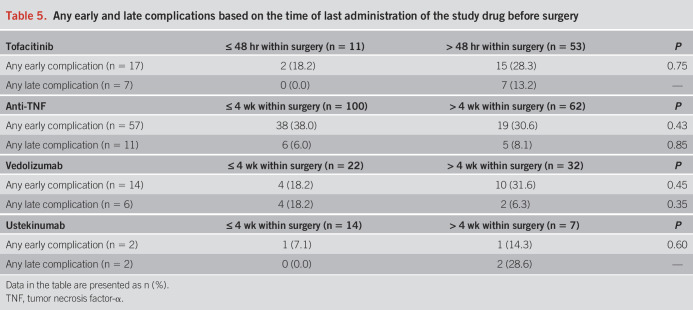
Any early and late complications based on the time of last administration of the study drug before surgery

In the multivariate model, urgent colectomy provided a higher risk of any early (OR 1.92, 95% CI 1.06–3.48, *P* = 0.03) complications. However, a subanalysis of separate complications showed that urgent colectomy increased only the risk of early hospital readmissions (OR 4.79, 95% CI 1.12–20.58, *P* = 0.04), and early redo surgery (OR 7.49, 95% CI 1.17–47.85, *P* = 0.03), with no influence of the other covariates included in the model on late complications. A steroid dose higher than the equivalent of 20 mg of oral prednisone increased the risk of any early complications (OR 1.96, 95% CI 1.08–3.57, *P* = 0.03), early SSC (OR 2.03, 95% CI 1.01–4.09, *P* = 0.048), and early redo surgery (OR 7.52, 95% CI 1.42–39.82, *P* = 0.02). Active smoking at surgery was an independent risk factor for developing at least one late complication (OR 4.67, 95% CI 1.48–14.77, *P* < 0.01). Regarding the type of surgery, laparoscopy resulted protective for any early complication (OR 0.54, 95% CI 0.29–1.00, *P* = 0.05), early infections (OR 0.39, 95% CI 0.18–0.85, *P* = 0.02), and late readmissions to hospital (OR 0.34, 95% CI 0.12–1.00, *P* = 0.05), whereas immediate IPAA increased the risk of early redo of surgery (OR 26.28, 95% CI 3.13-220.95, *P* < 0.01 for proctocolectomy + IPAA + ileostomy, and OR 81.70, 95% CI 1.22–5,463.98, *P* = 0.04 for one-stage proctocolectomy + IPAA, against total colectomy + ileostomy as reference category). Finally, a higher body mass index reduced the risk of early rehospitalization (OR 0.76, 95% CI 0.61–0.96, *P* = 0.02). Most importantly, the class of drug did not influence the risk of any early or late complications in the multivariate analysis, except for anti-TNF resulting protective for early readmission to hospital, although with similar rates between groups and very few events (see Supplementary Tables S4–S9, http://links.lww.com/AJG/D177).

## DISCUSSION

The impact of preoperative exposure to biologics on postoperative complications has been widely investigated in the literature. Despite initial controversies, it is now accepted that the occurrence of postoperative complications is more commonly related to the severity of IBD *per se* and the surgical circumstances rather than the use (and the type) of biologics (19). Accordingly, the PUCCINI study reported a similar early infection rate (within 30 days of surgery) in a cohort of 947 patients with IBD either treated with infliximab or unexposed to infliximab before surgery (18% vs 20%, *P* = 0.47) (13). In our study, the occurrence of early infectious complications in the anti-TNF group was similar to that of the PUCCINI study (22.8%, when combining infections and sepsis).

To date, little is known about the safety of tofacitinib in the surgical setting of total colectomy in patients with UC, although data of clinical trials in UC have showed similar concerns to traditional biologics and few additional red flags (4). Nevertheless, only one retrospective study on 53 patients with UC has analyzed the postcolectomy outcomes of tofacitinib exposure (11). The conclusion of this study was that tofacitinib might be generally safe, carrying an overall postsurgical infectious complication risk of 11%. However, concerns regarding an increased risk of postoperative VTE were raised, with 13% prevalence of these events at 90 days (11). However, the small sample size and the absence of comparison with other preoperative target therapies represented the 2 major weaknesses of this study, together with the exposure to multiple immunosuppressive drugs in the 12 weeks before surgery in 26% of patients (11). In our cohort, no significant differences between tofacitinib, anti-TNF, vedolizumab, and ustekinumab were found in terms of any early and late complications, early and late infections, and early and late sepsis.

Regarding non–anti-TNF biologics, the infection rate for vedolizumab and ustekinumab that was observed in our cohort is comparable with that reported in other recent studies (20–22). Taken together, these data are in line with the reported similar safety profile of tofacitinib compared with biologics in patients with UC (23).

The occurrence of SSC (including anastomotic leaks, SSI, and internal abscess at the site of anastomosis) was of particular interest in our study because it can significantly increase patient morbidity and postpone hospital discharge (24). No differences were reported in the rate of SSC, hospital readmissions, and redo surgery at both timepoints when comparing the exposure with the 4 different groups of immunosuppressive drugs. Because of the black box warning coming from the licensing authorities, particular attention was also given to the rate of VTE after colectomy. Surprisingly, significantly higher rates of early VTE in the anti-TNF group and of late VTE in the vedolizumab group were shown, whereas no VTE were reported for the 64 patients in the tofacitinib group. More particularly, all 9 early VTE were in the anti-TNF group, and all 2 late VTE were in the vedolizumab group. This result may be due to the more severely active course of the disease in patients in the anti-TNF group (that also presented an increased use of high dose of steroids, as high as 35%), which is correlated with a higher risk of VTE (25). However, the small number of events might have overestimated the rate differences of these outcomes at the univariate analysis. Notably, prophylactic LMWH was homogeneously distributed among groups.

In the multivariate analysis, none of the major outcomes was significantly influenced by the drug class, including tofacitinib. This result not only confirms the comparable safety of biologics in the preoperative setting but also provides positive feedback regarding the use of tofacitinib in this context. In an evolving scenario of increasing therapeutic options, it might occur that surgery is delayed in favor of attempting more medical treatments. However, delayed surgery can increase morbidity, hospital stay, and costs (26), which should also be considered in the general assessment of the disease. In line with the common knowledge that undergoing colectomy in emergency setting carries high mortality and morbidity (27–30), our data suggest that surgery should not be delayed. Accordingly, the patients of our cohort undergoing urgent surgery presented an increased risk of any early (1.9-fold) complications, when compared with elective colectomy.

The type of surgery also impacts the outcome of colectomy. Laparoscopic surgery offers a reduced risk of short-term complications, incisional hernias, and adhesions (31–33). On the other hand, there are conflicting data regarding the superiority of a 3-stage vs 1- or 2-stage approach in terms of postoperative complications (34–36), with international guidelines suggesting to prefer a 3-stage approach in patients under steroids or biologics and to avoid one stage approach in patients receiving anti-TNFs (37). We found an independent higher risk of redo surgery within 30 days in patients who underwent proctocolectomy + IPAA + ileostomy and one-stage proctocolectomy + IPAA compared with patients who underwent a total colectomy + ileostomy, although the number of operative stages did not influence any other complication category.

Current guidelines suggest to rapidly taper or stop steroids before surgery because of an increased risk of early infections and pouch-specific complications and to postpone pouch construction when this is not possible (37). In our study, a steroid dose higher than the equivalent of 20 mg of oral prednisone was found to increase the risk of any early complications, early SSC, and early redo surgery, which was in line with the current literature on the matter (19).

Finally, we found a 4.7-fold increased risk of any late complications in active smokers at the time of surgery, in line with current literature regarding an increased postoperative morbidity in active smokers undergoing abdominal surgery (38–40).

To the best of our knowledge, this is the first study assessing the safety of preoperative exposure to tofacitinib in comparison with biologics in patients with UC undergoing colectomy. The 4-group design, compared with a head-to-head design, allowed us a more granular comparison between immunosuppressive drugs with different mechanisms of action.

On the other hand, we also acknowledge some limitations. First, the retrospective design, which led to missing data, although information regarding clinical activity, endoscopy, and laboratory tests were present in at least 80% of the population. Moreover, despite the multicenter effort, the cohort was not sufficiently large to perform a propensity score matching and simulate the effect of randomization; however, homogeneity and multivariate analysis were performed to minimize the risk of biases. Similarly, the low number of patients included in the ustekinumab group may have led to selection bias.

Another issue concerns the short half-life of tofacitinib, as the 4-week window that we used might be considered too wide to impact the effect of this drug on surgical outcomes. However, in our opinion, this window reflects the frequent real-life clinical practice of patients with medically refractory disease undergoing surgery after treatment with tofacitinib, which was the main focus of our study. Moreover, the exposure window for the main analysis was based on the existing literature in the field (11), to make our data comparable and homogeneous for future meta-analyses. In addition, subanalyses with shorter exposure windows for both tofacitinib and biologics were also run, without showing any significant differences in terms of any complications. Finally, we reported a large observational time of 18 years, which could have led to biases because of unmeasured, surgery-related factors. However, stratification by year groups and sensitivity analysis to restrict to patients operated in the more recent years did not show meaningful differences from the original model. Moreover, the main surgery-related variables potentially influencing postoperative outcomes were included as covariates of our analysis (i.e., laparoscopy vs laparotomy, urgent vs elective surgery, and number of surgery stages).

In conclusion, our data demonstrated that tofacitinib and biologics have a similar rate of adverse events in the postoperative setting of patients undergoing colectomy for medically refractory UC. Urgent surgery and high doses of steroids before surgery were confirmed to be associated with a higher risk of early surgical complications. Larger and prospective studies are necessary to confirm these findings.

## CONFLICTS OF INTEREST

**Guarantor of the article:** Flavio Caprioli, MD, PhD.

**Specific author contributions:** G.D.: designed the study. G.D. and T.I.: analyzed the results and wrote the manuscript. All authors acquired data, interpreted the results, revised the article critically, and approved the final version to be submitted. F.C.: supervised and acted as guarantor of the article.

**Financial support:** None to report.

**Potential competing interests:** G.D. reports speaker fees from Alfasigma, Janssen, Novartis, Pfizer, Takeda, and has served in advisory board for Celltrion Healthcare and Pfizer F. Castiglione received lecture fees and served in advisory board for AbbVie, Biogen, Galapagos, Janssen, Pfizer, and Takeda. S.F. reports consultancy fees and/or advisory board for Galapagos, Janssen, Pfizer, and Takeda. E.V.S. has served as speaker for AbbVie, Agave, AGPharma, Alfasigma, Aurora Pharma, CaDiGroup, Celltrion, Dr Falk, EG Stada Group, Fenix Pharma, Fresenius Kabi, Galapagos, Janssen, JB Pharmaceuticals, Innovamedica/Adacyte, Malesci, Mayoly Biohealth, Omega Pharma, Pfizer, Reckitt Benckiser, Sandoz, SILA, Sofar, Takeda, Tillotts, and Unifarco; has served as consultant for AbbVie, Agave, Alfasigma, Biogen, Bristol-Myers Squibb, Celltrion, Diadema Farmaceutici, Dr. Falk, Fenix Pharma, Fresenius Kabi, Janssen, JB Pharmaceuticals, Merck & Co, Nestlè, Reckitt Benckiser, Regeneron, Sanofi, SILA, Sofar, Synformulas GmbH, Takeda, and Unifarco; he received research support from Pfizer, Reckitt Benckiser, SILA, Sofar, Unifarco, and Zeta Farmaceutici. D.G.R. reports speaker fees or advisory board services for Biogen, Celltrion Healthcare, Galapagos, Janssen, and Takeda. AnAr has served on advisory board for Galapagos. P.E. received lecture fees and travel educational grants from Bristol-Myers Squibb, Ferring, Pfizer, and Takeda. A.V. reports consultant fees from Pfizer. C.V. received consultancy and lecture fees from: AbbVie, Galapagos, Janssen, Pfizer, and Takeda. F. Caprioli served as consultant to AbbVie, Biogen, Bristol-Myers Squibb, Celgene, Eli-Lilly, Ferring, Galapagos, Gilead, Janssen, Lionhealth, MSD, Mundipharma, Nestlè, Pfizer, Roche, and Takeda; he received lecture fees from AbbVie, Allergy Therapeutics, Biogen, Ferring, Janssen, Pfizer, Sandoz, Takeda, Tillotts Pharma, and unrestricted research grants from AbbVie, Celltrion, Giuliani, MSD, Pfizer, Sofar, and Takeda. J.G. has served as speaker, consultant, and advisory member for or has received research funding from AbbVie, Biogen, Casen Fleet, Celgene/Bristol-Myers, Chiesi, Dr. Falk Pharma, Eli Lilly, Faes Farma, Ferring, Gebro Pharma, Gilead/Galapagos, Janssen, Kern Pharma, MSD, Mylan, Norgine, Otsuka Pharmaceutical, Pfizer, Roche, Sandoz, Shire Pharmaceuticals, Takeda, Tillotts Pharma, and Vifor Pharma. The other authors have no conflict of interest to declare.

**Data availability statement:** The data underlying this article will be shared on reasonable request to the corresponding author.

**Study Highlights**

**WHAT IS KNOWN**

✓ Patients with ulcerative colitis taking immunosuppressive drugs are at risk of colectomy.
✓ The postoperative safety of the preoperative exposure to tofacitinib is poorly documented.

**WHAT IS NEW HERE**

✓ Tofacitinib is as safe as biologics regarding the occurrence of postoperative complications.
✓ No warning signals concerning venous thromboembolic events were reported with tofacitinib.
✓ Urgent colectomy and high dose of steroids before surgery were confirmed as independent risk factors for postoperative complications.

## Supplementary Material
