# Supplementary material for: Rates of Adverse Events in Patients With Ulcerative Colitis Undergoing Colectomy During Treatment With Tofacitinib vs Biologics: A Multicenter Observational Study
Source: Am J Gastroenterol. 2024 Feb 2;119(8):1525–35. doi: 10.14309/ajg.0000000000002676 (PMC11288395; doi:10.14309/ajg.0000000000002676)
Supplement: Supplementary file 1 [file acg-119-1525-s001.docx]

**Supplementary tables**

**Table S1. Sensitivity analysis for shortened exposure windows (7 days for tofacitinib and 8 weeks for biologics)**

|  | **Tofacitinib (n = 22)** | **Anti-TNF (n = 148)** | **Vedolizumab (n = 46)** | **Ustekinumab (n = 20)** | **Total (n = 236)** | **P** |
| --- | --- | --- | --- | --- | --- | --- |
| **ANY EARLY COMPLICATIONS** | 7 (31.8%) | 53 (35.8%) | 13 (28.3%) | 2 (10.0%) | 75 (31.8%) | 0.13 |
| **EARLY INFECTIONS** | 5 (22.7%) | 22 (14.9%) | 7 (15.2%) | 0 (0.0%) | 34 (14.4%) | 0.20 |
| **EARLY SEPSIS** | 1 (4.5%) | 12 (8.1%) | 0 (0.0%) | 0 (0.0%) | 13 (5.5%) | 0.12 |
| **EARLY SSC** | 1 (4.5%) | 31 (20.9%) | 9 (19.6%) | 2 (10.0%) | 43 (18.2%) | 0.22 |
| **EARLY VTE** | 0 (0.0%) | 8 (5.4%) | 0 (0.0%) | 0 (0.0%) | 8 (3.4%) | 0.18 |
| **EARLY RE-HOSPITALISATIONS** | 1 (4.5%) | 14 (9.5%) | 3 (6.5%) | 1 (5.0%) | 19 (8.1%) | 0.76 |
| **EARLY REDO OF THE SURGERY** | 1 (4.5%) | 13 (8.8%) | 1 (2.2%) | 2 (10.0%) | 17 (7.2%) | 0.43 |
| **ANY LATE COMPLICATIONS** | 0 (0.0%) | 10 (6.8%) | 6 (13.0%) | 2 (10.0%) | 18 (7.6%) | 0.26 |
| **LATE INFECTIONS** | 0 (0.0%) | 3 (2.0%) | 1 (2.2%) | 1 (5.0%) | 5 (2.1%) | 0.73 |
| **LATE SEPSIS** | 0 (0.0%) | 0 (0.0%) | 0 (0.0%) | 0 (0.0%) | 0 (0.0%) | NA |
| **LATE SSC** | 0 (0.0%) | 3 (2.0%) | 2 (4.3%) | 1 (5.0%) | 6 (2.5%) | 0.61 |
| **LATE VTE** | 0 (0.0%) | 0 (0.0%) | 2 (4.3%) | 0 (0.0%) | 2 (0.8%) | **0.04** |
| **LATE RE-HOSPITALISATIONS** | 0 (0.0%) | 6 (4.1%) | 4 (8.7%) | 1 (5.0%) | 11 (4.7%) | 0.41 |
| **LATE REDO OF THE SURGERY** | 0 (0.0%) | 1 (0.7%) | 1 (2.2%) | 0 (0.0%) | 2 (0.8%) | 0.71 |

n (%); NA = not applicable; SSC = surgical site complications; TNF = tumour necrosis factor α; VTE = venous thromboembolic event

**Table S2. Sensitivity analysis for patients operated in the period 2018-2023**

|  | **Tofacitinib (n = 45)** | **Anti-TNF (n = 106)** | **Vedolizumab (n = 33)** | **Ustekinumab (n = 12)** | **Total (n = 196)** | **P** |
| --- | --- | --- | --- | --- | --- | --- |
| **ANY EARLY COMPLICATIONS** | 13 (28.9%) | 39 (36.8%) | 8 (24.2%) | 0 (0.0%) | 60 (30.6%) | 0.05 |
| **EARLY INFECTIONS** | 7 (15.6%) | 16 (15.1%) | 3 (9.1%) | 0 (0.0%) | 26 (13.3%) | 0.42 |
| **EARLY SEPSIS** | 3 (6.7%) | 11 (10.4%) | 0 (0.0%) | 0 (0.0%) | 14 (7.1%) | 0.16 |
| **EARLY SSC** | 6 (13.3%) | 22 (20.8%) | 6 (18.2%) | 0 (0.0%) | 34 (17.3%) | 0.27 |
| **EARLY VTE** | 0 (0.0%) | 5 (4.7%) | 0 (0.0%) | 0 (0.0%) | 5 (2.6%) | 0.23 |
| **EARLY RE-HOSPITALISATIONS** | 5 (11.1%) | 11 (10.4%) | 4 (12.1%) | 0 (0.0%) | 20 (10.2%) | 0.67 |
| **EARLY REDO OF THE SURGERY** | 3 (6.7%) | 11 (10.4%) | 0 (0.0%) | 0 (0.0%) | 14 (7.1%) | 0.16 |
| **ANY LATE COMPLICATIONS** | 5 (11.1%) | 9 (8.5%) | 3 (9.1%) | 0 (0.0%) | 17 (8.7%) | 0.69 |
| **LATE INFECTIONS** | 0 (0.0%) | 2 (1.9%) | 1 (3.0%) | 0 (0.0%) | 3 (1.5%) | 0.69 |
| **LATE SEPSIS** | 1 (2.2%) | 0 (0.0%) | 0 (0.0%) | 0 (0.0%) | 1 (0.5%) | 0.34 |
| **LATE SSC** | 3 (6.7%) | 3 (2.8%) | 1 (3.0%) | 0 (0.0%) | 7 (3.6%) | 0.60 |
| **LATE VTE** | 0 (0.0%) | 0 (0.0%) | 0 (0.0%) | 0 (0.0%) | 0 (0.0%) | NA |
| **LATE RE-HOSPITALISATIONS** | 3 (6.7%) | 6 (5.7%) | 3 (9.1%) | 0 (0.0%) | 12 (6.1%) | 0.72 |
| **LATE REDO OF THE SURGERY** | 2 (4.4%) | 1 (0.9%) | 1 (3.0%) | 0 (0.0%) | 4 (2.0%) | 0.50 |

n (%); NA = not applicable; SSC = surgical site complications; TNF = tumour necrosis factor α; VTE = venous thromboembolic event

**Table S3A. Univariate analysis by year of surgery (before/after 2018)**

|  | **< 2018 (n = 105)** | **≥ 2018 (n = 196)** | **Total (n = 301)** | **P** |
| --- | --- | --- | --- | --- |
| **ANY EARLY COMPLICATIONS** | 30 (28.6%) | 60 (30.6%) | 90 (29.9%) | 0.71 |
| **EARLY INFECTIONS** | 14 (13.3%) | 26 (13.3%) | 40 (13.3%) | 0.99 |
| **EARLY SEPSIS** | 3 (2.9%) | 14 (7.1%) | 17 (5.6%) | 0.13 |
| **EARLY SSC** | 19 (18.1%) | 34 (17.3%) | 53 (17.6%) | 0.87 |
| **EARLY VTE** | 4 (3.8%) | 5 (2.6%) | 9 (3.0%) | 0.54 |
| **EARLY RE-HOSPITALISATIONS** | 6 (5.7%) | 20 (10.2%) | 26 (8.6%) | 0.19 |
| **EARLY REDO OF THE SURGERY** | 6 (5.7%) | 14 (7.1%) | 20 (6.6%) | 0.64 |
| **ANY LATE COMPLICATIONS** | 9 (8.6%) | 17 (8.7%) | 26 (8.6%) | 0.98 |
| **LATE INFECTIONS** | 2 (1.9%) | 3 (1.5%) | 5 (1.7%) | 0.81 |
| **LATE SEPSIS** | 0 (0.0%) | 1 (0.5%) | 1 (0.3%) | 0.46 |
| **LATE SSC** | 4 (3.8%) | 7 (3.6%) | 11 (3.7%) | 0.92 |
| **LATE VTE** | 2 (1.9%) | 0 (0.0%) | 2 (0.7%) | 0.05 |
| **LATE RE-HOSPITALISATIONS** | 5 (4.8%) | 12 (6.1%) | 17 (5.6%) | 0.63 |
| **LATE REDO OF THE SURGERY** | 0 (0.0%) | 4 (2.0%) | 4 (1.3%) | 0.14 |

n (%);SSC = surgical site complications; VTE = venous thromboembolic event

**Table S3B. Univariate analysis by year groups**

|  | **2005-2010 (n = 12)** | **2011-2015 (n = 51)** | **2016-2020 (n = 134)** | **2021-2023 (n = 104)** | **Total (n = 301)** | **P** |
| --- | --- | --- | --- | --- | --- | --- |
| **ANY EARLY COMPLICATIONS** | 4 (33.3%) | 16 (31.4%) | 37 (27.6%) | 33 (31.7%) | 90 (29.9%) | 0.89 |
| **EARLY INFECTIONS** | 2 (16.7%) | 9 (17.6%) | 13 (9.7%) | 16 (15.4%) | 40 (13.3%) | 0.42 |
| **EARLY SEPSIS** | 1 (8.3%) | 0 (0.0%) | 7 (5.2%) | 9 (8.7%) | 17 (5.6%) | 0.17 |
| **EARLY SSC** | 2 (16.7%) | 10 (19.6%) | 24 (17.9%) | 17 (16.3%) | 53 (17.6%) | 0.97 |
| **EARLY VTE** | 0 (0.0%) | 0 (0.0%) | 8 (6.0%) | 1 (1.0%) | 9 (3.0%) | 0.06 |
| **EARLY RE-HOSPITALISATIONS** | 1 (8.3%) | 3 (5.9%) | 9 (6.7%) | 13 (12.5%) | 26 (8.6%) | 0.38 |
| **EARLY REDO OF THE SURGERY** | 1 (8.3%) | 4 (7.8%) | 6 (4.5%) | 9 (8.7%) | 20 (6.6%) | 0.60 |
| **ANY LATE COMPLICATIONS** | 0 (0.0%) | 5 (9.8%) | 9 (6.7%) | 12 (11.5%) | 26 (8.6%) | 0.40 |
| **LATE INFECTIONS** | 0 (0.0%) | 1 (2.0%) | 1 (0.7%) | 3 (2.9%) | 5 (1.7%) | 0.60 |
| **LATE SEPSIS** | 0 (0.0%) | 0 (0.0%) | 0 (0.0%) | 1 (1.0%) | 1 (0.3%) | 0.59 |
| **LATE SSC** | 0 (0.0%) | 2 (3.9%) | 5 (3.7%) | 4 (3.8%) | 11 (3.7%) | 0.92 |
| **LATE VTE** | 0 (0.0%) | 1 (2.0%) | 1 (0.7%) | 0 (0.0%) | 2 (0.7%) | 0.55 |
| **LATE RE-HOSPITALISATIONS** | 0 (0.0%) | 2 (3.9%) | 7 (5.2%) | 8 (7.7%) | 17 (5.6%) | 0.60 |
| **LATE REDO OF THE SURGERY** | 0 (0.0%) | 0 (0.0%) | 2 (1.5%) | 2 (1.9%) | 4 (1.3%) | 0.76 |

n (%); SSC = surgical site complications; VTE = venous thromboembolic event

**Table S4. Early infections**

| **Covariates** | | **YES**  ***n* = 40** | **NO**  ***n* = 261** | **Univariate** | **Multivariate** | | | |
| --- | --- | --- | --- | --- | --- | --- | --- | --- |
|  |  |  |  |  | **OR** | **95%CI** | | ***P*** |
| **FEMALES** [n (%)] | | 14 (35.0) | 109 (41.8) | 0.42 |  |  |  |  |
| **DISEASE EXTENSION** | **E1+ E2** [n (%)] | 5 (12.5) | 75 (28.7) | 0.09 | reference |  |  |  |
|  | **E3** [n (%)] | 35 (87.5) | 186 (71.3) |  | 2.25 | 0.82 | 6.18 | 0.11 |
| **AGE AT SURGERY** [years/10; median (IQR)] | | 39.8 (31.7-60.8) | 38.7 (28.2-53.9) | 0.33 |  |  |  |  |
| **DISEASE DURATION AT SURGERY** [years; median (IQR)] | | 2.9 (1.5-7.4) | 5.4 (2.4-10.9) | **0.02** | 0.95 | 0.88 | 1.01 | 0.11 |
| **SHORT DRUG-TO-SURGERY INTERVAL*** [n (%)] | | 34 (85.0) | 202 (77.4) | 0.28 |  |  |  |  |
| **DRUG EXPOSURE** [months; median (IQR)] | | 4.2 (1.6-15.2) | 4.3 (1.5-10.5) | 0.61 |  |  |  |  |
| **EXPERIENCED TO BIOLOGICS** [n (%)] | | 21 (52.5) | 162 (62.1) | 0.24 |  |  |  |  |
| **URGENT SURGERY** [n (%)] | | 23 (57.5) | 75 (28.7) | **< 0.01** | 1.92 | 0.87 | 4.21 | 0.11 |
| **LAPAROSCOPIC SURGERY** [n (%)] | | 23 (57.5) | 211 (80.8) | **< 0.01** | 0.39 | 0.18 | 0.85 | **0.02** |
| **TOTAL COLECTOMY AND ILEOSTOMY** [n (%)] | | 25 (62.5) | 148 (56.7) | 0.49 |  |  |  |  |
| **PROCTOCOLECTOMY + IPAA AND ILEOSTOMY** [n (%)] | | 15 (37.5) | 105 (40.2) |  |  |  |  |  |
| **ONE-STAGE PROCTOCOLECTOMY + IPAA** [n (%)] | | 0 (0.0) | 8 (3.1) |  |  |  |  |  |
| **STEROIDS > 20 mg USE** [n (%)] | | 17 (42.5) | 69 (26.4) | **0.04** | 1.28 | 0.59 | 2.80 | 0.53 |
| **ANTIBIOTICS PROPHYLAXIS** [n (%)] | | 25 (62.5) | 129 (49.4) | 0.12 |  |  |  |  |
| **LMWH PROPHYLAXIS** [n (%)] | | 22 (55.0) | 161 (61.7) | 0.42 |  |  |  |  |
| **ACTIVE SMOKERS** [n (%)] | | 3 (7.5) | 16 (6.1) | 0.74 |  |  |  |  |
| **DIABETES MELLITUS** [n (%)] | | 3 (7.5) | 18 (6.9) | 1.00 |  |  |  |  |
| **PERIPHERAL VASCULAR DISEASE** [n (%)] | | 0 (0.0) | 4 (1.5) | 0.96 |  |  |  |  |
| **CHARLSON COMORBIDITY INDEX** [median (IQR)] | | 0.0 (0.0-3-0) | 0.0 (0.0-1.0) | 0.18 |  |  |  |  |
| **BODY MASS INDEX (*n* = 260)** [median (IQR)] | | 21.9 (18.6-23.4) | 21.5 (19.1-24.8) | 0.54 |  |  |  |  |
| **ALBUMIN (*n* = 193)** [g/dL; median (IQR)] | | 3.2 (2.8-3.8) | 3.5 (2.9-3.9) | 0.25 |  |  |  |  |
| **C-REACTIVE PROTEIN (*n* = 269)** [mg/dL; median (IQR)] | | 2.2 (1.1-5.8) | 2.0 (0.7-5.2) | 0.66 |  |  |  |  |
| **TOFACITINIB** [n (%)] | | 9 (22.5) | 55 (21.1) | 0.31 | reference |  |  |  |
| **ANTI-TNF** [n (%)] | | 24 (60.0) | 138 (52.9) |  | 0.71 | 0.29 | 1.75 | 0.46 |
| **VEDOLIZUMAB** [n (%)] | | 7 (17.5) | 47 (18.0) |  | 0.80 | 0.26 | 2.48 | 0.70 |
| **USTEKINUMAB** [n (%)] | | 0 (0.0) | 21 (8.0) |  | 0.00 | 0.00 | . | 1.00 |

CI = confidence interval; IPAA = ileal pouch-anal anastomosis; IQR = interquartile range; LMWH = low molecular weight heparin; OR = odds ratio; TNF = tumour necrosis factor α

* patients who had their last drug dose within a shorter time before surgery (i.e., tofacitinib ≤ 7 days; biologics ≤ 8 weeks)

**Table S5. Early sepsis**

| **Covariates** | | **YES**  ***n* = 17** | **NO**  ***n* = 284** | **Univariate** | **Multivariate** | | | |
| --- | --- | --- | --- | --- | --- | --- | --- | --- |
|  |  |  |  |  | **OR** | **95%CI** | | ***P*** |
| **FEMALES** [n (%)] | | 9 (52.9) | 114 (40.1) | 0.30 |  |  |  |  |
| **DISEASE EXTENSION** | **E1+ E2** [n (%)] | 5 (29.4) | 75 (26.4) | 0.76 |  |  |  |  |
|  | **E3** [n (%)] | 12 (70.6) | 209 (73.6) |  |  |  |  |  |
| **AGE AT SURGERY** [years/10; median (IQR)] | | 44.6 (28.7-52.6) | 38.6 (28.2-54.1) | 0.71 |  |  |  |  |
| **DISEASE DURATION AT SURGERY** [years; median (IQR)] | | 2.5 (2.2-6.2) | 5.2 (2.2.-10.6) | 0.05 | 0.94 | 0.83 | 1.06 | 0.30 |
| **SHORT DRUG-TO-SURGERY INTERVAL*** [n (%)] | | 13 (76.5) | 223 (78.5) | 0.84 |  |  |  |  |
| **DRUG EXPOSURE** [months; median (IQR)] | | 4.6 (2.7-9.2) | 4.3 (1.5-11.1) | 0.83 |  |  |  |  |
| **EXPERIENCED TO BIOLOGICS** [n (%)] | | 10 (58.8) | 173 (60.9) | 0.86 |  |  |  |  |
| **URGENT SURGERY** [n (%)] | | 12 (70.6) | 86 (30.3) | **< 0.01** | 2.48 | 0.74 | 8.32 | 0.14 |
| **LAPAROSCOPIC SURGERY** [n (%)] | | 11 (64.7) | 223 (78.5) | 0.18 |  |  |  |  |
| **TOTAL COLECTOMY AND ILEOSTOMY** [n (%)] | | 8 (47.1) | 165 (58.1) | 0.37 |  |  |  |  |
| **PROCTOCOLECTOMY + IPAA AND ILEOSTOMY** [n (%)] | | 8 (47.1) | 112 (39.4) |  |  |  |  |  |
| **ONE-STAGE PROCTOCOLECTOMY + IPAA** [n (%)] | | 1 (5.9) | 7 (2.5) |  |  |  |  |  |
| **STEROIDS > 20 mg USE** [n (%)] | | 9 (52.9) | 77 (27.1) | **0.02** | 1.82 | 0.61 | 5.42 | 0.28 |
| **ANTIBIOTICS PROPHYLAXIS** [n (%)] | | 10 (58.8) | 144 (50.7) | 0.52 |  |  |  |  |
| **LMWH PROPHYLAXIS** [n (%)] | | 13 (76.5) | 170 (59.9) | 0.27 |  |  |  |  |
| **ACTIVE SMOKERS** [n (%)] | | 2 (11.8) | 17 (6.0) | 0.66 |  |  |  |  |
| **DIABETES MELLITUS** [n (%)] | | 0 (0.0) | 21 (7.4) | 0.50 |  |  |  |  |
| **PERIPHERAL VASCULAR DISEASE** [n (%)] | | 0 (0.0) | 4 (1.4) | 1.00 |  |  |  |  |
| **CHARLSON COMORBIDITY INDEX** [median (IQR)] | | 0.0 (0.0-3.0) | 0.0 (0.0-2.0) | 0.27 |  |  |  |  |
| **BODY MASS INDEX (*n* = 260)** [median (IQR)] | | 21.5 (18.4-22.1) | 21.6 (19.1-24.7) | 0.46 |  |  |  |  |
| **ALBUMIN (*n* = 193)** [g/dL; median (IQR)] | | 3.2 (2.1-3.9) | 3.5 (2.9-3.9) | 0.32 |  |  |  |  |
| **C-REACTIVE PROTEIN (*n* = 269)** [mg/dL; median (IQR)] | | 5.6 (1.3-11.7) | 2.0 (0.7-4.8) | **0.05** | 1.05 | 0.97 | 1.13 | 0.24 |
| **TOFACITINIB** [n (%)] | | 4 (23.5) | 60 (21.1) | 0.10 | reference |  |  |  |
| **ANTI-TNF** [n (%)] | | 13 (76.5) | 149 (52.5) |  | 0.87 | 0.25 | 3.02 | 0.83 |
| **VEDOLIZUMAB** [n (%)] | | 0 (0.0) | 54 (19.0) |  | 0.00 | 0.00 | . | 1.00 |
| **USTEKINUMAB** [n (%)] | | 0 (0.0) | 21 (7.4) |  | 0.00 | 0.00 | . | 1.00 |

CI = confidence interval; IPAA = ileal pouch-anal anastomosis; IQR = interquartile range; LMWH = low molecular weight heparin; OR = odds ratio; TNF = tumour necrosis factor α

* patients who had their last drug dose within a shorter time before surgery (i.e., tofacitinib ≤ 7 days; biologics ≤ 8 weeks)

**Table S6. Early surgical site complications**

| **Covariates** | | **YES**  ***n* = 53** | **NO**  ***n* = 248** | **Univariate** | **Multivariate** | | | |
| --- | --- | --- | --- | --- | --- | --- | --- | --- |
|  |  |  |  |  | **OR** | **95%CI** | | ***P*** |
| **FEMALES** [n (%)] | | 18 (34.0) | 105 (42.3) | 0.26 |  |  |  |  |
| **DISEASE EXTENSION** | **E1+ E2** [n (%)] | 19 (35.8) | 61 (24.6) | 0.07 | reference |  |  |  |
|  | **E3** [n (%)] | 34 (64.2) | 187 (75.4) |  | 1.98 | 1.00 | 3.90 | 0.05 |
| **AGE AT SURGERY** [years/10; median (IQR)] | | 44.6 (28.6-61.4) | 38.4 (28.2-52.5) | 0.09 | 1.01 | 0.99 | 1.04 | 0.33 |
| **DISEASE DURATION AT SURGERY** [years; median (IQR)] | | 3.1 (2.1-9.8) | 5.4 (2.3-10.5) | 0.19 |  |  |  |  |
| **SHORT DRUG-TO-SURGERY INTERVAL*** [n (%)] | | 43 (81.1) | 193 (77.8) | 0.60 |  |  |  |  |
| **DRUG EXPOSURE** [months; median (IQR)] | | 3.5 (0.5-9.2) | 4.5 (0.6-11.3) | 0.32 |  |  |  |  |
| **EXPERIENCED TO BIOLOGICS** [n (%)] | | 28 (52.8) | 155 (62.5) | 0.19 |  |  |  |  |
| **URGENT SURGERY** [n (%)] | | 24 (45.3) | 74 (29.8) | **0.03** | 1.64 | 0.82 | 3.28 | 0.16 |
| **LAPAROSCOPIC SURGERY** [n (%)] | | 38 (71.7) | 196 (79.0) | 0.24 |  |  |  |  |
| **TOTAL COLECTOMY AND ILEOSTOMY** [n (%)] | | 30 (56.6) | 143 (57.7) | 0.89 |  |  |  |  |
| **PROCTOCOLECTOMY + IPAA AND ILEOSTOMY** [n (%)] | | 22 (41.5) | 98 (39.5) |  |  |  |  |  |
| **ONE-STAGE PROCTOCOLECTOMY + IPAA** [n (%)] | | 1 (1.9) | 7 (2.8) |  |  |  |  |  |
| **STEROIDS > 20 mg USE** [n (%)] | | 24 (45.3) | 62 (25.0) | **< 0.01** | 2.03 | 1.01 | 4.09 | **0.048** |
| **ANTIBIOTICS PROPHYLAXIS** [n (%)] | | 35 (66.0) | 119 (48.0) | **0.02** | 0.56 | 0.28 | 1.11 | 0.10 |
| **LMWH PROPHYLAXIS** [n (%)] | | 37 (69.8) | 146 (58.9) | 0.14 |  |  |  |  |
| **ACTIVE SMOKERS** [n (%)] | | 6 (11.3) | 13 (5.2) | 0.10 | 0.52 | 0.17 | 1.62 | 0.26 |
| **DIABETES MELLITUS** [n (%)] | | 4 (7.5) | 17 (6.9) | 0.86 |  |  |  |  |
| **PERIPHERAL VASCULAR DISEASE** [n (%)] | | 1 (1.9) | 3 (1.2) | 1.00 |  |  |  |  |
| **CHARLSON COMORBIDITY INDEX** [median (IQR)] | | 0.2 (0.2-2.0) | 0.0 (0.0-1.0) | 0.05 | 1.10 | 0.83 | 1.46 | 0.50 |
| **BODY MASS INDEX (*n* = 260)** [median (IQR)] | | 21.2 (19.2-24.0) | 21.6 (19.0-24.7) | 0.72 |  |  |  |  |
| **ALBUMIN (*n* = 193)** [g/dL; median (IQR)] | | 3.2 (2.2-3.8) | 3.5 (2.9-3.9) | 0.32 |  |  |  |  |
| **C-REACTIVE PROTEIN (*n* = 269)** [mg/dL; median (IQR)] | | 2.8 (1.8-5.7) | 2.0 (1.7-5.1) | 0.26 |  |  |  |  |
| **TOFACITINIB** [n (%)] | | 8 (15.1) | 56 (22.6) | 0.40 |  |  |  |  |
| **ANTI-TNF** [n (%)] | | 33 (62.3) | 129 (52.0) |  | 1.39 | 0.58 | 3.36 | 0.46 |
| **VEDOLIZUMAB** [n (%)] | | 10 (18.9) | 44 (17.7) |  | 1.20 | 0.41 | 3.53 | 0.74 |
| **USTEKINUMAB** [n (%)] | | 2 (3.8) | 19 (7.7) |  | 0.53 | 0.09 | 3.08 | 0.48 |

CI = confidence interval; IPAA = ileal pouch-anal anastomosis; IQR = interquartile range; LMWH = low molecular weight heparin; OR = odds ratio; TNF = tumour necrosis factor α

* patients who had their last drug dose within a shorter time before surgery (i.e., tofacitinib ≤ 7 days; biologics ≤ 8 weeks)

**Table S7. Early re-hospitalisations**

| **Covariates** | | **YES**  ***n* = 26** | **NO**  ***n* = 275** | **Univariate** | **Multivariate** | | | |
| --- | --- | --- | --- | --- | --- | --- | --- | --- |
|  |  |  |  |  | **OR** | **95%CI** | | ***P*** |
| **FEMALES** [n (%)] | | 6 (23.1) | 117 (42.5) | 0.05 | 4.95 | 1.14 | 21.62 | **0.03** |
| **DISEASE EXTENSION** | **E1+ E2** [n (%)] | 9 (34.6) | 71 (25.8) | 0.37 |  |  |  |  |
|  | **E3** [n (%)] | 17 (65.4) | 204 (74.2) |  |  |  |  |  |
| **AGE AT SURGERY** [years/10; median (IQR)] | | 29.3 (24.0-46.0) | 39.7 (28.8-54.9) | **0.03** | 0.98 | 0.92 | 1.03 | 0.42 |
| **DISEASE DURATION AT SURGERY** [years; median (IQR)] | | 4.2 (2.2-8.8) | 5.1 (2.2-10.5) | 0.69 |  |  |  |  |
| **SHORT DRUG-TO-SURGERY INTERVAL*** [n (%)] | | 19 (73.1) | 217 (78.9) | 0.49 |  |  |  |  |
| **DRUG EXPOSURE** [months; median (IQR)] | | 2.1 (1.2-7.0) | 4.7 (1.6-11.5) | 0.08 | 1.01 | 1.00 | 1.02 | **0.01** |
| **EXPERIENCED TO BIOLOGICS** [n (%)] | | 15 (57.7) | 168 (61.1) | 0.73 |  |  |  |  |
| **URGENT SURGERY** [n (%)] | | 15 (57.7) | 83 (30.2) | **0.00** | 4.79 | 1.12 | 20.58 | **0.04** |
| **LAPAROSCOPIC SURGERY** [n (%)] | | 15 (57.7) | 219 (79.6) | **0.01** | 0.75 | 0.17 | 3.31 | 0.71 |
| **TOTAL COLECTOMY AND ILEOSTOMY** [n (%)] | | 12 (46.2) | 161 (58.5) | 0.22 |  |  |  |  |
| **PROCTOCOLECTOMY + IPAA AND ILEOSTOMY** [n (%)] | | 14 (53.8) | 106 (38.5) |  |  |  |  |  |
| **ONE-STAGE PROCTOCOLECTOMY + IPAA** [n (%)] | | 0 (0.0) | 8 (2.9) |  |  |  |  |  |
| **STEROIDS > 20 mg USE** [n (%)] | | 14 (53.8) | 72 (26.2) | **0.00** | 2.66 | 0.65 | 10.99 | 0.18 |
| **ANTIBIOTICS PROPHYLAXIS** [n (%)] | | 18 (69.2) | 136 (49.5) | 0.05 | 3.96 | 0.86 | 18.27 | 0.08 |
| **LMWH PROPHYLAXIS** [n (%)] | | 15 (57.7) | 168 (61.1) | 0.73 |  |  |  |  |
| **ACTIVE SMOKERS** [n (%)] | | 2 (7.7) | 17 (6.2) | 0.76 |  |  |  |  |
| **DIABETES MELLITUS** [n (%)] | | 4 (15.4) | 17 (6.2) | 0.17 |  |  |  |  |
| **PERIPHERAL VASCULAR DISEASE** [n (%)] | | 0 (0.0) | 4 (1.5) | 0.54 |  |  |  |  |
| **CHARLSON COMORBIDITY INDEX** [median (IQR)] | | 0.0 (0.0-1.0) | 0.0 (0.0-2.0) | 0.25 |  |  |  |  |
| **BODY MASS INDEX (*n* = 260)** [median (IQR)] | | 19.0 (17.0-21.0) | 21.9 (19.2-24.8) | **0.00** | 0.76 | 0.61 | 0.96 | **0.02** |
| **ALBUMIN (*n* = 193)** [g/dL; median (IQR)] | | 3.3 (2.9-3.6) | 3.5 (2.9-3.9) | 0.25 |  |  |  |  |
| **C-REACTIVE PROTEIN (*n* = 269)** [mg/dL; median (IQR)] | | 1.8 (0.6-4.9) | 2.1 (0.7-5.4) | 0.85 |  |  |  |  |
| **TOFACITINIB** [n (%)] | | 7 (26.9) | 57 (20.7) | 0.82 | reference |  |  |  |
| **ANTI-TNF** [n (%)] | | 14 (53.8) | 148 (53.8) |  | 0.15 | 0.04 | 0.67 | **0.01** |
| **VEDOLIZUMAB** [n (%)] | | 4 (15.4) | 50 (18.2) |  | 0.10 | 0.01 | 1.17 | 0.07 |
| **USTEKINUMAB** [n (%)] | | 1 (3.8) | 20 (7.3) |  | 0.00 | 0.00 | . | 1.00 |

CI = confidence interval; IPAA = ileal pouch-anal anastomosis; IQR = interquartile range; LMWH = low molecular weight heparin; OR = odds ratio; TNF = tumour necrosis factor α

* patients who had their last drug dose within a shorter time before surgery (i.e., tofacitinib ≤ 7 days; biologics ≤ 8 weeks)

**Table S8. Late re-hospitalisations**

| **Covariates** | | **YES**  ***n* = 17** | **NO**  ***n* = 284** | **Univariate** | **Multivariate** | | | |
| --- | --- | --- | --- | --- | --- | --- | --- | --- |
|  |  |  |  |  | **OR** | **95%CI** | | ***P*** |
| **FEMALES** [n (%)] | | 6 (35.3) | 117 (41.2) | 0.63 |  |  |  |  |
| **DISEASE EXTENSION** | **E1+ E2** [n (%)] | 8 (47.1) | 72 (25.4) | 0.08 | reference |  |  |  |
|  | **E3** [n (%)] | 9 (52.9) | 212 (74.6) |  | 0.37 | 0.13 | 1.05 | 0.06 |
| **AGE AT SURGERY** [years/10; median (IQR)] | | 41.1 (31.3-49.6) | 38.6 (27.9-54.2) | 0.89 |  |  |  |  |
| **DISEASE DURATION AT SURGERY** [years; median (IQR)] | | 3.1 (2.4-5.9) | 5.2 (2.2-10.5) | 0.22 |  |  |  |  |
| **SHORT DRUG-TO-SURGERY INTERVAL*** [n (%)] | | 11 (64.7) | 225 (79.2) | 0.16 |  |  |  |  |
| **DRUG EXPOSURE** [months; median (IQR)] | | 2.7 (1.5-6.0) | 4.5 (1.5-11.4) | 0.29 |  |  |  |  |
| **EXPERIENCED TO BIOLOGICS** [n (%)] | | 12 (70.6) | 171 (60.2) | 0.40 |  |  |  |  |
| **URGENT SURGERY** [n (%)] | | 7 (41.2) | 91 (32.0) | 0.44 |  |  |  |  |
| **LAPAROSCOPIC SURGERY** [n (%)] | | 10 (58.8) | 224 (78.9) | 0.05 | 0.34 | 0.12 | 1.00 | **0.05** |
| **TOTAL COLECTOMY AND ILEOSTOMY** [n (%)] | | 6 (35.3) | 167 (58.8) | 0.06 | reference |  |  |  |
| **PROCTOCOLECTOMY + IPAA AND ILEOSTOMY** [n (%)] | | 10 (58.8) | 110 (38.7) |  | 2.70 | 0.93 | 7.87 | 0.07 |
| **ONE-STAGE PROCTOCOLECTOMY + IPAA** [n (%)] | | 1 (5.9) | 7 (2.5) |  | 3.91 | 0.39 | 39.35 | 0.25 |
| **STEROIDS > 20 mg USE** [n (%)] | | 5 (29.4) | 81 (28.5) | 0.94 |  |  |  |  |
| **ANTIBIOTICS PROPHYLAXIS** [n (%)] | | 8 (47.1) | 146 (51.4) | 0.73 |  |  |  |  |
| **LMWH PROPHYLAXIS** [n (%)] | | 8 (47.1) | 175 (61.6) | 0.23 |  |  |  |  |
| **ACTIVE SMOKERS** [n (%)] | | 1 (5.9) | 18 (6.3) | 0.94 |  |  |  |  |
| **DIABETES MELLITUS** [n (%)] | | 1 (5.9) | 20 (7.0) | 0.86 |  |  |  |  |
| **PERIPHERAL VASCULAR DISEASE** [n (%)] | | 0 (0.0) | 4 (1.4) | 0.62 |  |  |  |  |
| **CHARLSON COMORBIDITY INDEX** [median (IQR)] | | 0.0 (0.0-1.0) | 0.0 (0.0-2.0) | 0.96 |  |  |  |  |
| **BODY MASS INDEX (*n* = 260)** [median (IQR)] | | 21.0 (18.6-26.7) | 21.6 (19.0-24.5) | 0.94 |  |  |  |  |
| **ALBUMIN (*n* = 193)** [g/dL; median (IQR)] | | 3.6 (2.9-4.1) | 3.5 (2.9-3.9) | 0.55 |  |  |  |  |
| **C-REACTIVE PROTEIN (*n* = 269)** [mg/dL; median (IQR)] | | 2.3 (0.7-5.6) | 2.0 (0.7-5.4) | 0.95 |  |  |  |  |
| **TOFACITINIB** [n (%)] | | 5 (29.4) | 59 (20.8) | 0.70 | reference |  |  |  |
| **ANTI-TNF** [n (%)] | | 7 (41.2) | 155 (54.6) |  | 0.43 | 0.12 | 1.52 | 0.19 |
| **VEDOLIZUMAB** [n (%)] | | 4 (23.5) | 50 (17.6) |  | 0.84 | 0.20 | 3.51 | 0.81 |
| **USTEKINUMAB** [n (%)] | | 1 (5.9) | 20 (7.0) |  | 0.49 | 0.05 | 4.60 | 0.53 |

CI = confidence interval; IPAA = ileal pouch-anal anastomosis; IQR = interquartile range; LMWH = low molecular weight heparin; OR = odds ratio; TNF = tumour necrosis factor α

* patients who had their last drug dose within a shorter time before surgery (i.e., tofacitinib ≤ 7 days; biologics ≤ 8 weeks)

**Table S9. Early redo of surgery**

| **Covariates** | | **YES**  ***n* = 20** | **NO**  ***n* = 281** | **Univariate** | **Multivariate** | | | |
| --- | --- | --- | --- | --- | --- | --- | --- | --- |
|  |  |  |  |  | **OR** | **95%CI** | | ***P*** |
| **FEMALES** [n (%)] | | 6 (30.0) | 117 (41.6) | 0.31 |  |  |  |  |
| **DISEASE EXTENSION** | **E1+ E2** [n (%)] | 10 (50.0) | 70 (24.9) | **0.02** | reference |  |  |  |
|  | **E3** [n (%)] | 10 (50.0) | 211 (75.1) |  | 0.09 | 0.02 | 0.47 | **< 0.01** |
| **AGE AT SURGERY** [years/10; median (IQR)] | | 37.3 (26.0-46.5) | 38.9 (28.5-54.1) | 0.46 |  |  |  |  |
| **DISEASE DURATION AT SURGERY** [years; median (IQR)] | | 3.1 (2.2-5.4) | 5.3 (2.2-10.5) | 0.18 |  |  |  |  |
| **SHORT DRUG-TO-SURGERY INTERVAL*** [n (%)] | | 17 (85.0) | 219 (77.9) | 0.65 |  |  |  |  |
| **DRUG EXPOSURE** [months; median (IQR)] | | 2.6 (0.8-5.2) | 4.7 (1.5-11.5) | **0.04** | 0.89 | 0.78 | 1.02 | 0.09 |
| **EXPERIENCED TO BIOLOGICS** [n (%)] | | 12 (60.0) | 171 (60.9) | 0.94 |  |  |  |  |
| **URGENT SURGERY** [n (%)] | | 13 (65.0) | 85 (30.2) | **< 0.01** | 7.49 | 1.17 | 47.85 | **0.03** |
| **LAPAROSCOPIC SURGERY** [n (%)] | | 15 (75.0) | 219 (77.9) | 0.76 |  |  |  |  |
| **TOTAL COLECTOMY AND ILEOSTOMY** [n (%)] | | 5 (25.0) | 168 (59.8) | **< 0.01** | reference |  |  |  |
| **PROCTOCOLECTOMY + IPAA AND ILEOSTOMY** [n (%)] | | 14 (70.0) | 106 (37.7) |  | 26.28 | 3.13 | 220.95 | **< 0.01** |
| **ONE-STAGE PROCTOCOLECTOMY + IPAA** [n (%)] | | 1 (5.0) | 7 (2.5) |  | 81.70 | 1.22 | 5463.98 | **0.04** |
| **STEROIDS > 20 mg USE** [n (%)] | | 14 (70.0) | 72 (25.6) | **< 0.01** | 7.52 | 1.42 | 39.82 | **0.02** |
| **ANTIBIOTICS PROPHYLAXIS** [n (%)] | | 15 (75.0) | 139 (49.5) | **0.05** | 2.21 | 0.28 | 17.30 | 0.45 |
| **LMWH PROPHYLAXIS** [n (%)] | | 15 (75.0) | 168 (59.8) | 0.18 |  |  |  |  |
| **ACTIVE SMOKERS** [n (%)] | | 1 (5.0) | 18 (6.4) | 0.80 |  |  |  |  |
| **DIABETES MELLITUS** [n (%)] | | 1 (5.0) | 20 (7.1) | 0.72 |  |  |  |  |
| **PERIPHERAL VASCULAR DISEASE** [n (%)] | | 0 (0.0) | 4 (1.4) | 0.59 |  |  |  |  |
| **CHARLSON COMORBIDITY INDEX** [median (IQR)] | | 0.0 (0.0-2.0) | 0.0 (0.0-2.0) | 0.54 |  |  |  |  |
| **BODY MASS INDEX (*n* = 260)** [median (IQR)] | | 19.1 (18.0-22.0) | 21.8 (19.2-24.7) | 0.12 |  |  |  |  |
| **ALBUMIN (*n* = 193)** [g/dL; median (IQR)] | | 3.1 (2.5-3.6) | 3.5 (2.9-3.9) | 0.09 | 0.70 | 0.22 | 2.27 | 0.55 |
| **C-REACTIVE PROTEIN (*n* = 269)** [mg/dL; median (IQR)] | | 5.7 (1.8-11.7) | 2.0 (0.7-4.7) | **< 0.01** | 1.04 | 0.93 | 1.16 | 0.52 |
| **TOFACITINIB** [n (%)] | | 4 (20.0) | 60 (21.4) | 0.43 | reference |  |  |  |
| **ANTI-TNF** [n (%)] | | 13 (65.0) | 149 (53.0) |  | 0.68 | 0.11 | 4.34 | 0.69 |
| **VEDOLIZUMAB** [n (%)] | | 1 (5.0) | 53 (18.9) |  | 0.00 | 0.00 | . | 1.00 |
| **USTEKINUMAB** [n (%)] | | 2 (10.0) | 19 (6.8) |  | 3.31 | 0.30 | 36.47 | 0.33 |

CI = confidence interval; IPAA = ileal pouch-anal anastomosis; IQR = interquartile range; LMWH = low molecular weight heparin; OR = odds ratio; TNF = tumour necrosis factor α

* patients who had their last drug dose within a shorter time before surgery (i.e., tofacitinib ≤ 7 days; biologics ≤ 8 weeks)
